# Granzyme A inhibition reduces inflammation and increases survival during abdominal sepsis

**DOI:** 10.7150/thno.49288

**Published:** 2021-01-30

**Authors:** Marcela Garzón-Tituaña, José L Sierra-Monzón, Laura Comas, Llipsy Santiago, Tatiana Khaliulina-Ushakova, Iratxe Uranga-Murillo, Ariel Ramirez-Labrada, Elena Tapia, Elena Morte-Romea, Sonia Algarate, Ludovic Couty, Eric Camerer, Phillip I Bird, Cristina Seral, Pilar Luque, José R Paño-Pardo, Eva M Galvez, Julián Pardo, Maykel Arias

**Affiliations:** 1 Fundación Instituto de Investigación Sanitaria Aragón (IIS Aragón), Biomedical Research Centre of Aragón (CIBA), 50009, Zaragoza, Spain.; 2 Instituto de Carboquímica ICB-CSIC, 50018, Zaragoza, Spain.; 3 Aragon I+D Foundation (ARAID), 50018, Zaragoza, Spain.; 4 Nanoscience Institute of Aragon (INA), University of Zaragoza, 50018, Zaragoza, Spain.; 5 Department of Biochemistry and Molecular and Cell Biology and Department of Microbiology, Preventive Medicine and Public Health, University of Zaragoza, 50009, Zaragoza, Spain.; 6 Hospital Clínico Universitario Lozano Blesa, 50009, Zaragoza, Spain.; 7 Animal Unit, University of Zaragoza, 50009, Zaragoza, Spain.; 8 INSERM U970, Paris Cardiovascular Research Centre, Université de Paris, 75015, Paris, France.; 9 Department of Biochemistry and Molecular Biology, Biomedicine Discovery Institute, Monash University, 3800, Clayton VIC, Australia.

**Keywords:** Granzyme A, inflammation, peritonitis, sepsis, cecal ligation and puncture.

## Abstract

**Aims:** Peritonitis is one of the most common causes of sepsis, a serious syndrome characterized by a dysregulated systemic inflammatory response. Recent evidence suggests that Granzyme A (GzmA), a serine protease mainly expressed by NK and T cells, could act as a proinflammatory mediator and could play an important role in the pathogenesis of sepsis. This work aims to analyze the role and the therapeutic potential of GzmA in the pathogenesis of peritoneal sepsis.

**Methods:** The level of extracellular GzmA as well as GzmA activity were analyzed in serum from healthy volunteers and patients with confirmed peritonitis and were correlated with the Sequential Organ Failure Assessment (SOFA) score. Peritonitis was induced in C57Bl/6 (WT) and GzmA^-/-^ mice by cecal ligation and puncture (CLP). Mice were treated intraperitoneally with antibiotics alone or in combination serpinb6b, a specific GzmA inhibitor, for 5 days. Mouse survival was monitored during 14 days, levels of some proinflammatory cytokines were measured in serum and bacterial load and diversity was analyzed in blood and spleen at different times.

**Results:** Clinically, elevated GzmA was observed in serum from patients with abdominal sepsis suggesting that GzmA plays an important role in this pathology. In the CLP model GzmA deficient mice, or WT mice treated with an extracellular GzmA inhibitor, showed increased survival, which correlated with a reduction in proinflammatory markers in both serum and peritoneal lavage fluid. GzmA deficiency did not influence bacterial load in blood and spleen and GzmA did not affect bacterial replication in macrophages *in vitro,* indicating that GzmA has no role in bacterial control. Analysis of GzmA in lymphoid cells following CLP showed that it was mainly expressed by NK cells. Mechanistically, we found that extracellular active GzmA acts as a proinflammatory mediator in macrophages by inducing the TLR4-dependent expression of IL-6 and TNFα.

**Conclusions:** Our findings implicate GzmA as a key regulator of the inflammatory response during abdominal sepsis and provide solid evidences about its therapeutic potential for the treatment of this severe pathology.

## Introduction

Peritonitis is an inflammation of the peritoneum, usually following infection of the serous membrane that covers the abdominal cavity and the organs contained therein 1. Peritonitis is classified as primary, secondary or tertiary depending on disease severity and effect on the different organs 2. Primary peritonitis is associated with undamaged intra-abdominal cavity organs. The most frequent forms are spontaneous bacterial peritonitis, associated with advanced liver disease (infected ascites), and infection in patients undergoing peritoneal dialysis. Secondary peritonitis is an infection in the peritoneal cavity due to a perforation of the gastrointestinal tract by ulceration, ischemia or obstruction; a post-surgical infection or a closed or penetrating trauma. Tertiary peritonitis is a persistent intra-abdominal infection after at least 48 hours of adequate therapy of primary or secondary peritonitis 2. Peritonitis is a leading cause of sepsis 1, a serious syndrome characterized by a dysregulated systemic inflammatory response 3, 4.

Granzymes (Gzms) are a family of serine proteases that, together with perforin, constitute the main components of the cytotoxic lymphocyte granule exocytosis pathway. Gzms are classified according to their cleavage specificity. Five Gzms in humans (A, B, H, K, and M) and ten in mice (A, B, C, D, E, F, G, K, M and N) have been described 5. The granule exocytosis pathway is a specialized form of intracellular protein delivery by which lymphocytes release perforin and Gzms into infected or compromised cells. Perforin creates short-lived pores in the plasma membrane of target cells allowing the passage of Gzms into the cytosol of target cells where they carry out their effector functions 6. Gzms can also be released into the extracellular milieu where they regulate different extracellular processes independently of their ability to induce cell death 5, 7. Traditionally, it was assumed that all Gzms acted as cytotoxic proteases. However, recent evidence suggests that only GzmB has clear cytotoxic capacity, while the cytotoxicity of others such as GzmA and GzmK is controversial 6, 8-12.

It has been recognised that some Gzms like GzmA may act as pro-inflammatory mediators contributing to the pathophysiology of different inflammatory disorders including rheumatoid and viral arthritis 13, 14, colitis 15, endotoxicosis 16, 17 or bacterial sepsis 18, 19. Furthermore, *in vitro* studies have reported that GzmA induces the expression of pro-inflammatory cytokines in several cell types including monocytes, macrophages, endothelial and epithelial cells and fibroblasts 16, 20-23. However, the role of GzmA in abdominal sepsis, the second most common form of sepsis in humans, is not known.

This study aimed to analyse the involvement of GzmA in abdominal sepsis, and gain insight into its therapeutic potential. We examined serum samples from patients with intraabdominal sepsis; and investigated the role of GzmA in sepsis using the Cecum Ligation and Puncture (CLP) mouse model, which best mimics the septic response during human peritonitis 24.

## Results

### Extracellular GzmA is increased in patients undergoing abdominal sepsis

**Figure 1:**
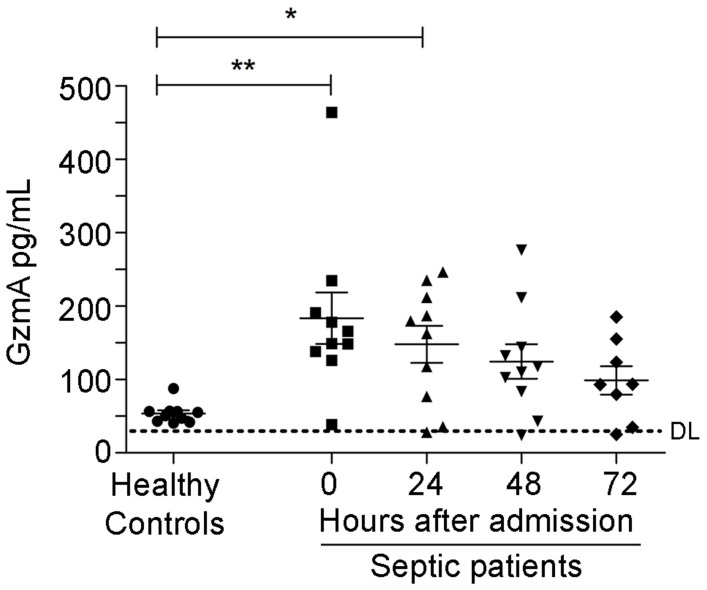
** Increased levels of extracellular GzmA are observed in patients with abdominal sepsis.** Serum levels of GzmA were analysed by ELISA in healthy donors (n = 10) and compared with GzmA levels from patients with a diagnosis of abdominal sepsis (n = 10) at diagnosis (time 0 = disease onset) and during the first 72 h. Statistical analyses were performed by one-way ANOVA test with Bonferroni's post-test *P < 0.05; **P < 0.01. DL (ELISA Detection Limit).

We analysed the levels of GzmA in serum from patients with abdominal sepsis, who were diagnosed with peritonitis with a SOFA (Sepsis related Organ Failure Assessment) score >2, and compared with the levels in serum from healthy donors. Patient characteristics are included in Table 1. There were 6 males and 4 females, the medium age was 76 years and 3 patients deceased after 30 days. Patients with peritoneal sepsis presented elevated levels of GzmA at time of diagnosis (time 0) and for 72 h thereafter that were significantly above healthy donor levels both diagnosis and after 24 h (Figure 1). This shows that serum GzmA is elevated during abdominal sepsis and suggests that extracellular GzmA could be involved in its pathogenesis.

### *GZMA* deficient mice are protected from CLP

Once we had observed increased levels of extracellular GzmA in patients with abdominal sepsis, we decided to analyse the relevance of these findings by testing the role of GzmA in the CLP mouse model, one of the murine models that better mimics the complex septic response in human abdominal sepsis 24. A severe sepsis CLP protocol was applied to WT and *GZMA*^-/-^ mice as indicated in methods. In order to mimic the clinical management of septic patients, a broad-spectrum antimicrobial treatment was administered starting six hours after CLP, and survival monitored for 14 days.

**Figure 2:**
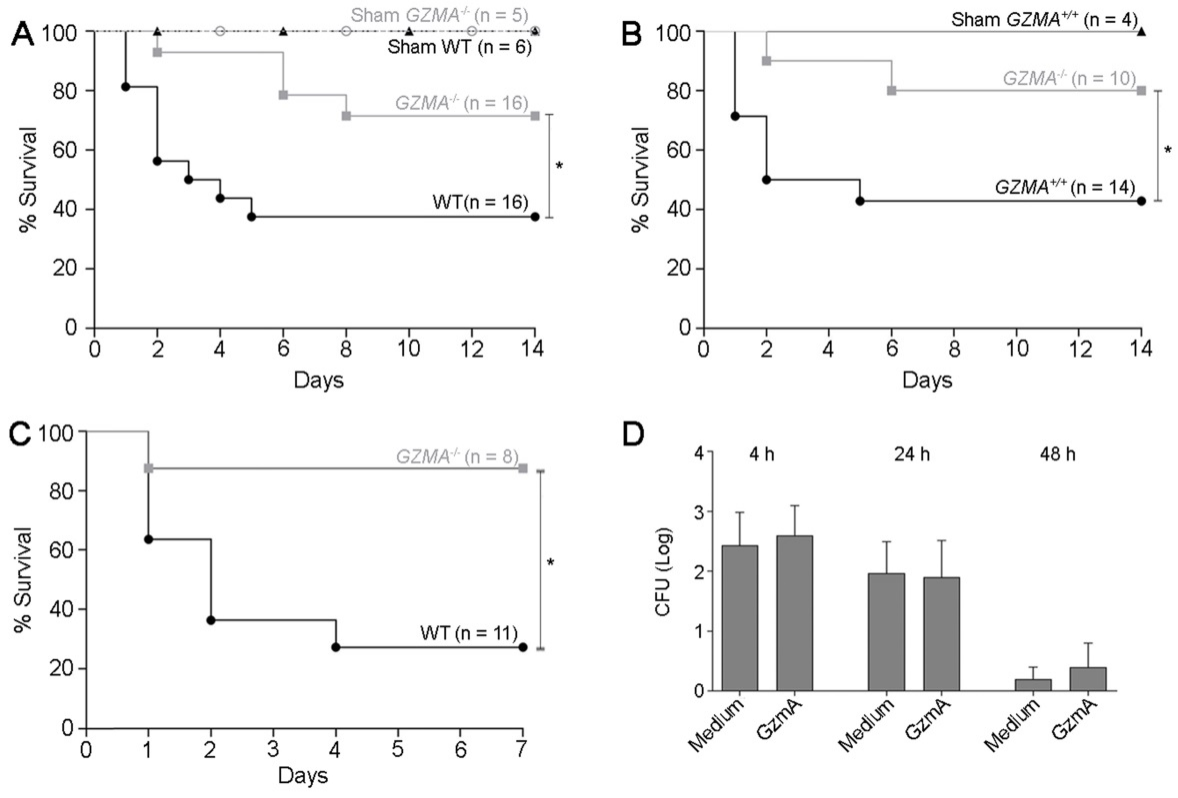
** The absence of granzyme A increases survival in mice during CLP and *E. coli*-induced sepsis**. Sepsis was induced by CLP in WT and *GZMA*^-/-^ mice **(A)** and GZMA^-/-^ and GZMA^+/+^ littermates **(B)** as described in materials and methods. After 6 h, a mixture of antibiotics, ceftriaxone (30 mg/kg) + Metronidazole (12.5 mg/kg) was administered i.p. every 24 h for 5 days. WT and *GZMA*^-/-^ sham operated mice underwent the same procedure but without the ligation and puncture of the cecum. Survival was monitored during 14 days. The data correspond to the indicated number of biological replicates (individual mice) from three independent experiments. Statistical analyse was performed using logrank and Gehan-Wilcoxon test. *p < 0.05. **(C)** WT and GZMA^-/-^ mice were infected i.p. with 2 x 10^8^ CFU of *E. coli* as described in materials and methods. The data corresponds to the indicated number of biological replicates from two independent experiments. Statistical analyse was performed using logrank and Gehan-Wilcoxon test. *p < 0.05. **(D)** An *in vitro* analysis was performed to determine the capacity of GzmA in the control of bacterial pathogens. M1 macrophages were differentiated as described in material and methods. Macrophages were incubated with GZMA (300 nM), infected with *E. coli* (MOI 1:100) and incubated for 4, 24 and 48 h at 37 °C. After incubation time cells were lysed and the number of CFU was determined. Data are presented as mean ± SEM from 3 independent experiments.

As shown in Figure 2A survival of *GZMA*^-/-^ mice was significantly higher than WT controls. Less than 40% of WT mice survived CLP, while more than 70% of *GZMA*^-/-^ mice were still alive after 2 weeks. As expected, all sham mice survived, which confirms that the surgical technique was performed effectively. When control littermates (Figure 2B) were used a similar result was found, confirming discarding that minimal differences in genetic background between WT and GZMA^-/-^ mice could influence the results. These results indicate that GzmA plays an important role during severe abdominal sepsis induced by CLP* in vivo*. To confirm the role of GZMA in peritoneal sepsis we employed *E. coli*-induced sepsis as additional model. As shown in Figure 2C, GZMA^-/-^ mice survived significantly longer than WT mice confirming the results obtained in CLP model. Finally, incubation of GzmA with macrophages infected with *E. coli* did not potentiate inhibition of bacterial replication (Figure 2D), indicating that the results obtained *in vivo* are not influenced by changes in bacterial replication in the absence of GZMA (see also Figure 4).

### *GZMA* deficient mice show reduced levels of proinflammatory cytokines during abdominal sepsis

**Figure 3:**
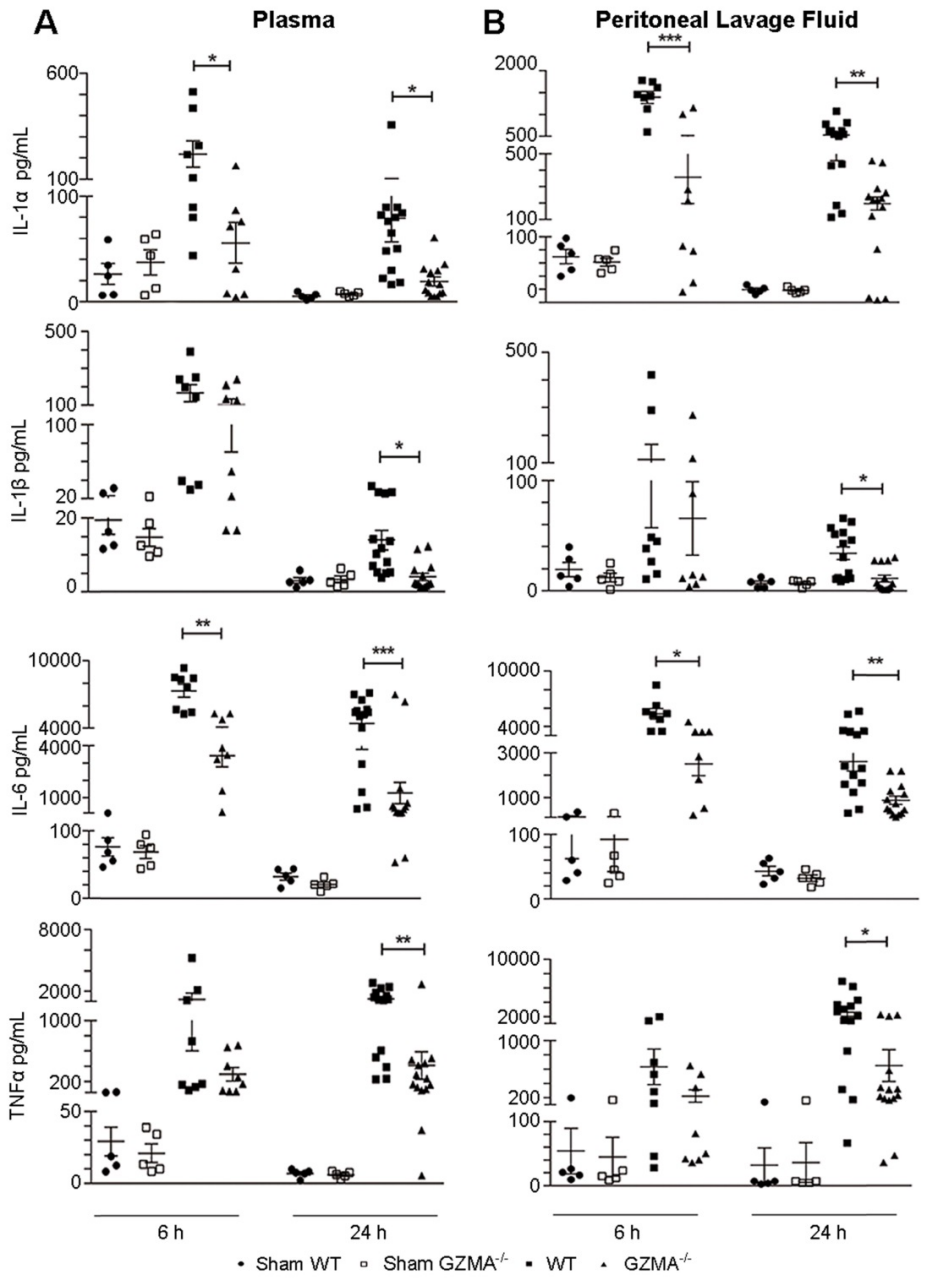
** The absence of granzyme A reduces the level of proinflammatory cytokines during sepsis induced by CLP**. Sepsis was induced by CLP in WT and *GZMA*^-/-^ mice as described in materials and methods. After 6 h, a mixture of antibiotics, ceftriaxone (30 mg/kg) + metronidazole (12.5 mg/kg) was administered every 12 h. WT and *GZMA*^-/-^ sham operated mice underwent the same procedure but without CLP. After 6 or 24 h of sepsis induction, mice were sacrificed and the levels of IL-1α, IL-1β, TNFα and IL-6 in plasma and peritoneal lavage fluids were determined by ELISA. Data are presented as mean ± SEM of 5 (sham) or at least 8 (CLP) different biological replicates (individual mice) from 3 independent experiments. Statistical analysis was performed by one-way ANOVA test with Bonferroni's post-test *P < 0.05; **P < 0.01; ***P < 0.001.

Next, we analysed if the protection observed in *GZMA* deficient mice during sepsis induced by CLP correlated with reduced serum level of proinflammatory cytokines. Thus, we monitored the concentrations of IL-6, IL-1α, IL-1β and TNFα in plasma and peritoneal lavage fluid (Figure 3). *GZMA*^-/-^ mice showed significantly lower levels of all cytokines in both plasma and peritoneal lavage fluid after 6 and/or 24 h of CLP induction. It should be noted that although all cytokines were reduced in the biological fluids of *GZMA*^-/-^ mice after CLP, only IL-6 and IL-1α were significantly lower than controls at 6 h in both plasma and peritoneal fluid. After 24 h all cytokines, IL-6, IL-1α, IL-1β and TNFα, were significantly reduced in septic *GZMA*^-/-^ mice. This confirms that GzmA regulates the generation of proinflammatory cytokines *in vivo* during abdominal sepsis.

### The absence of GzmA does not compromise anti-bacterial control during CLP

**Figure 4:**
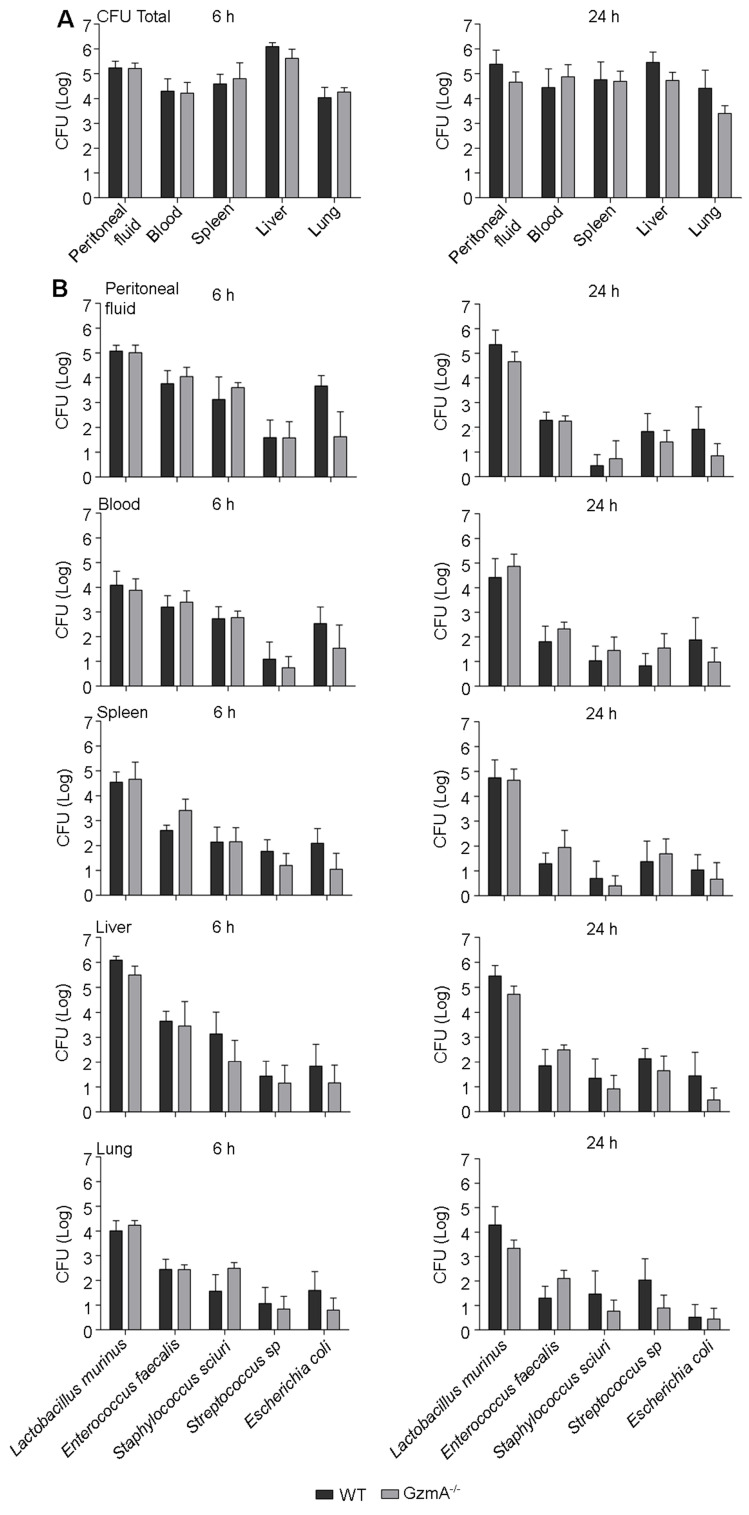
** GzmA is not involved in the control of bacterial pathogens during sepsis induced by CLP.** Sepsis was induced by CLP in WT and *GZMA*^-/-^ mice as described in materials and methods. After 6 h, a mixture of antibiotics, ceftriaxone (30 mg/kg) + Metronidazole (12.5 mg/kg) was administered i.p. every 12 h. After 6 and 24 h of sepsis induction, a group of mice were sacrificed and the total number of CFU from aerobic bacteria was determined in peritoneal fluids, blood, spleen liver and lung (**A**). The most frequent strains were identified by MALDI-TOF mass spectrometry and the number of CFU of these strains was determined in peritoneal lavage fluids, blood, spleen liver and lung (**B**). Data are presented as mean ± SEM from 5 biological replicates (individual mice) in each group.

*GZMA* deficiency reduces the generation of inflammatory cytokines *in vivo.* Thus, it is possible that the reduced inflammatory response affects efficient clearance of local and/or systemic bacterial infection after CLP. We analysed the total aerobic bacterial load in blood, spleen, liver, lungs and peritoneal fluid during CLP-induced sepsis. As shown in Figure 4A, WT and *GZMA*^-/-^ mice exhibited similar bacterial loads in peritoneal lavage fluid, blood, liver, spleen and lung after 6 and 24 h of sepsis induction. Although total bacterial counts were similar in both mouse strains, it is possible that the control of specific bacterial species is compromised in the absence of GzmA. Thus, we characterised the bacterial species presented in the different organs and fluids and quantified their individual level by MALDI-TOF mass spectrometry. The analysis showed that the most frequent species present were *Lactobacillus murinus*, *Enterococcus faecalis*, *Staphylococcus sciuri*, *Streptococcus sp* and *Escherichia coli.* The bacterial load of these species in WT and *GZMA*^-/-^ mice was similar in peritoneal fluids, blood, spleen, liver and lung, after 6 and 24 h of sepsis induction (Figure 4B). These results indicate that GzmA deficiency does not compromise control of aerobic bacteria during polymicrobial abdominal sepsis and together with figure 2D indicate that the effect of inflammatory effect of GzmA does not contribute to bacterial control.

### GzmA expression increases in NK cells during abdominal sepsis

**Figure 5:**
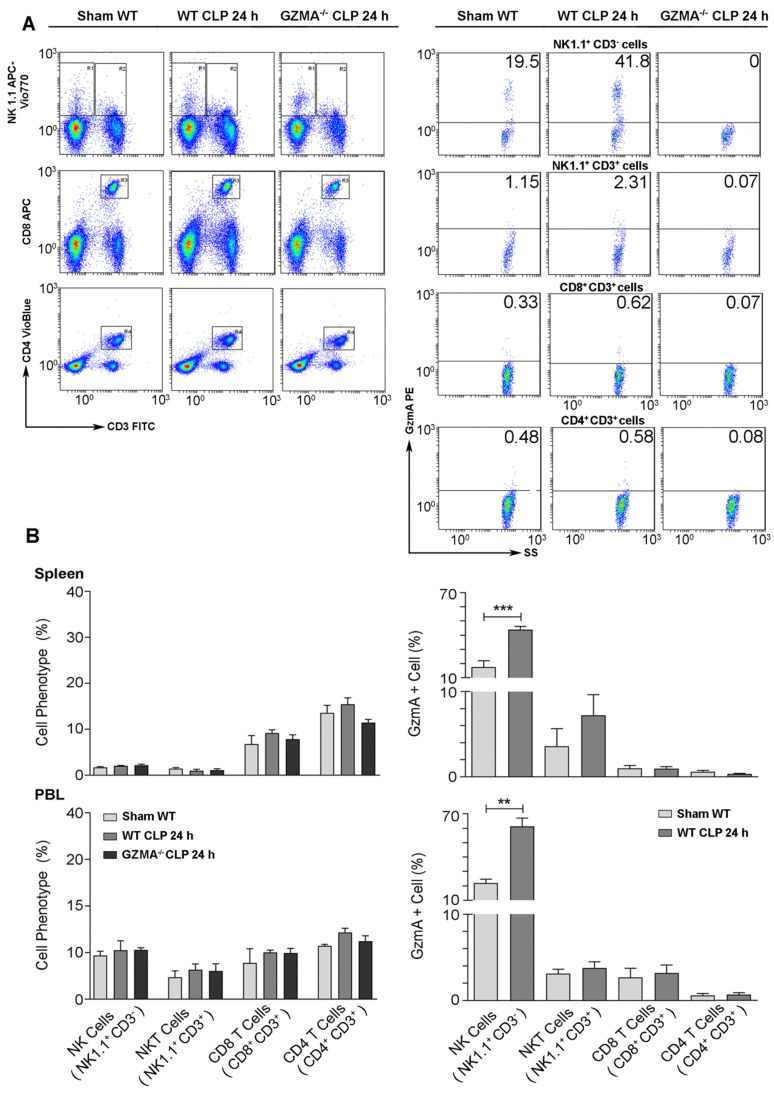
** GzmA expression is increased in NK cells from septic mice.** Sepsis was induced by CLP in WT and *GZMA*^-/-^ mice as described in materials and methods. Sham WT operated mice underwent the same procedure but without the ligation and puncture of the cecum. After 24 h mice were sacrificed and (A) the percentage of NK cells (NK1.1^+^CD3^-^), NKT cells (NK1.1^+^ CD3^+^), CD8^+^ T cells (CD8^+^ CD3^+^) and CD4^+^ T cells (CD4^+^, CD3^+^) and (B) the intracellular expression of GzmA on NK cells, NKT cells, CD8^+^ T cells and CD4^+^ T cells were analysed in splenocytes and PBLs by flow cytometry. A representative experiment of GzmA expression in spleen is shown via dot plot (**A**). Numbers show the cell percentage in each quadrant. Data in graphs represent the mean ± SEM of the percentage of GzmA positive cells of each phenotype (right) and the percentage of each subtype (left) from 2 independent experiments. (**B**) Statistical analysis was performed by unpaired student's t test. **p < 0.01, ***p < 0.001.

Once we confirmed that the absence of GzmA increases the survival of mice during abdominal CLP-induced sepsis, correlating with a reduced inflammatory response that did not compromise bacterial control, we decided to establish the cellular source of GzmA. We focused on the major cell populations known to express GzmA in blood and spleen 5: NK and T cells, including NKT cells. The gating strategy is summarised in Figure 5A. Cells were stained with NK1.1, CD3, CD4 and CD8 antibodies in order to distinguish NK cells (NK1.1^+^CD3^-^ cells) from the main T cell subsets, NKT (NK1.1^+^CD3^+^), CD4^+^ T (NK1.1^-^CD3^+^CD4^+^) and CD8^+^T (NK1.1^-^CD3^+^CD8^+^) cells. As shown in Figure 5A, the relative presence of these populations was not different between WT and *GZMA*^-/-^ mice confirming that the absence of GzmA does not influence these immune cell responses. GzmA expression was significantly enhanced in NK cells from septic mice in both spleen and peripheral blood. In contrast, the expression of GzmA in the main T cell subsets (NKT, CD8^+^ T or CD4^+^ T cells) was very low and did not increase during sepsis (Figure 5B).

### Therapeutic inhibition of GzmA with Serpinb6b increases survival and reduces inflammation during CLP

**Figure 6:**
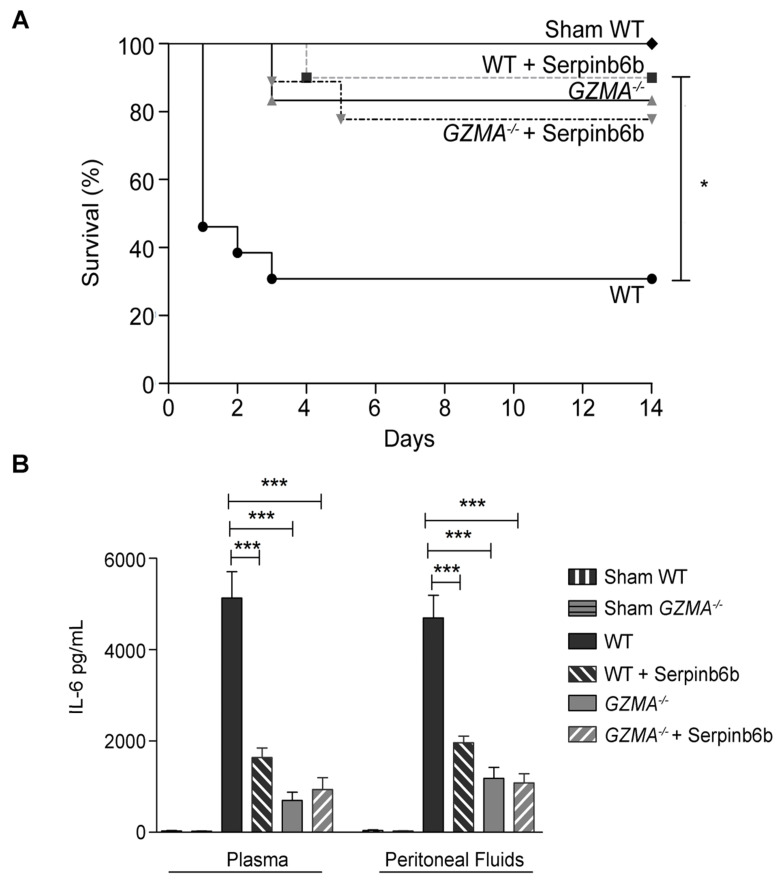
** Inhibition of extracellular GzmA improves sepsis outcome and reduces inflammation.** Sepsis was induced by CLP in B6 and *GZMA*^-/-^ mice as described in materials and methods. Immediately after surgery mice were treated with 40 µg of Serpinb6b in 100 µl of PBS (10 WT and 9 *GZMA^-/-^* mice). This treatment was repeated 6 h later and once a day during 5 days. Control mice received only 100 µl of PBS i.p. (13 WT and 6 *GZMA^-/-^* mice). After 6 h a mixture of antibiotics, ceftriaxone (30 mg/kg) + metronidazole (12.5 mg/kg) was administered i.p. once a day for 5 days. WT sham operated mice (4 WT mice) underwent the same procedure but without the ligation and puncture of the cecum. **(A)** Survival was monitored during 14 days. The data correspond to the indicated number of mice combined from two independent experiments. Statistical analyse was performed using logrank and Gehan-Wilcoxon test. *p < 0.05. **(B)** 24 h after sepsis induction mice were sacrificed and the levels of IL-6 in plasma and peritoneal fluids was determined by ELISA. Data are presented as mean ± SEM of 4 (Sham) or 6 (CLP) biological replicates from 2 independent experiments. Statistical analysis was performed by one-way ANOVA test with Bonferroni's post-test *P < 0.05; **P < 0.01; ***P < 0.001.

**Figure 7:**
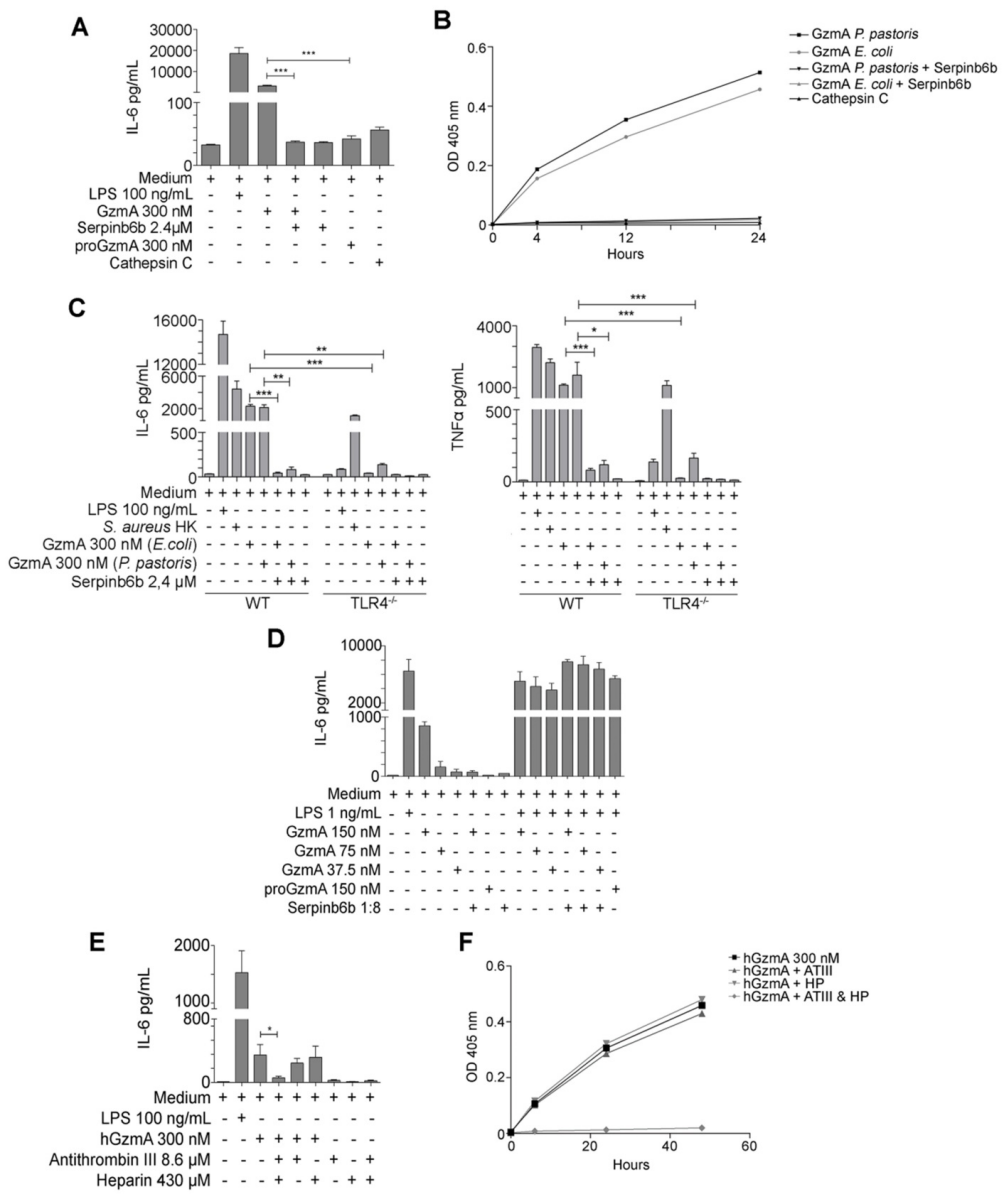
** Active extracellular GzmA induces the expression of IL-6 and TNFα in macrophages by a mechanism dependent of TLR4. (A)** WT bone marrow differentiated macrophages were stimulated with LPS 100 ng/mL, active and inactive GzmA produced in *E. coli*, GzmA inactivated with sepinb6b and cathepsin C. After 24 h of incubation, supernatant was collected to determine the levels of IL-6 by ELISA. **(B)** GzmA activity was confirmed by an activity essay incubating active and inactive GzmA produced in *E. coli*, GzmA inactivated with sepinb6b and cathepsin C with a specific mouse GzmA substrate. Optical density at 405 nm was measured after 4, 12 and 24 h. **(C)** Macrophages differentiated from WT or TLR4^-/-^ mouse bone marrow were stimulated with active GzmA (300 nM) produced in *E. coli* or in *P. pastoris*, GzmA inactivated with serpinb6b, LPS 100 ng/mL or *S. aureus* HK (1 x10^6^ CFU/mL). After 24 h of incubation, the supernatant was collected to determine the levels of IL-6 and TNFα by ELISA. Data are represented as the mean ± SEM of two independent experiments performed by duplicate. Statistical analyses were performed by one-way ANOVA test with Bonferroni's post-test, *P < 0.05, **P < 0.01; ***P < 0.001. **(D)** Macrophages were stimulated with LPS 1 ng/mL, GzmA (150 nM, 75 nM and 37,5 nM), inactive GzmA and serpinb6b. After 24 h incubation, supernatants were collected to determine the levels of IL-6 by ELISA. **(E)** Human monocytes were obtained as described in materials and methods. 5 x 10^3^ human monocytes were stimulated with LPS 100 ng/mL, human active GzmA and human GzmA inactivated with antithrombin III (ATIII) and heparin (HP). After 24 h incubation, supernatant was collected to determine levels of IL-6 by ELISA. Statistical analysis was performed by unpaired student's t test, ***P < 0.001. **(F)** Human GzmA activity was confirmed by an activity essay incubating active human GzmA (300 nM), human GzmA inactivated with antithrombin III (ATIII) and heparin (HP) with a specific human GzmA substrate. Optical density at 405 nm was measured after 4, 12 and 24 h.

Once the effect of GzmA absence was analysed using a genetically deficient model, we decided to further analyse the potential of using GzmA as therapeutic target to treat abdominal sepsis. To this aim we analysed the effect of a GzmA inhibitor on WT animals undergoing abdominal sepsis. We used serpinb6b, a recently described natural specific inhibitor of mouse GzmA, which does not inhibit other gzms 25. After sepsis induction a group of WT and *GZMA* deficient mice were treated with the inhibitor and survival was monitored during 14 days. As shown in Figure 6A, WT mice treated with antibiotics and serpinb6b showed a significant improvement in survival compared with WT mice treated with antibiotics only. Survival of septic WT mice treated with serpinb6b was similar to the survival of *GZMA* deficient mice. In contrast, the inhibitor did not have any effect on *GZMA* deficient mice suggesting that the effect observed in WT mice was specifically a consequence of GzmA inhibition. In parallel, a group of mice treated or not with the inhibitor were sacrificed and the level of IL-6 in plasma and peritoneal fluid was determined. Since all cytokines were reduced in GZMA^-/-^ mice after 24 h, we decided to focus only in IL-6 in WT mice treated with the inhibitor as a representative cytokine in order to confirm the results obtained in GZMA^-/-^ mice. In WT mice the therapeutic inhibition of GzmA significantly reduced the level of IL-6 in plasma and in peritoneal fluids. By contrast, the administration of serpinb6b in septic *GZMA^-/-^*mice did not reduce the levels of IL-6, again confirming the specificity of serpinb6b to inhibit GzmA (Figure 6B). The ability of serpinb6b to inhibit the enzymatic activity of GzmA was also confirmed (Figure 7). These results confirm that GzmA inhibition in septic WT mice mimics the effect observed in *GZMA* deficient mice and provide a proof of principle that inhibition of GzmA could be useful for the treatment of abdominal sepsis.

### The inflammatory response induced by GzmA on mouse macrophages depends on TLR4 expression

Increased levels of GzmA were found in patients with abdominal sepsis (Figure 1) and human GzmA is known to induce the generation of inflammatory cytokines in monocytes and macrophages 16, 26. Thus, in order to find out how extracellular GzmA contributes to abdominal sepsis, we analysed the generation of inflammatory cytokines in mouse macrophages *in vitro*. As shown in Figure 7A, mouse active GzmA significantly induced IL-6 expression in mouse macrophages. Inactive proGzmA or GzmA inhibited by Serpinb6b did not induce IL-6. As additional control we used cathepsin C the protease used to activate GzmA in the purification process. As expected, since cathepsin C is not active at pH higher than 6, this protease did not induce IL-6 production. The amount of LPS in the recombinant protein preparation was lower than 0.5 EU/μg. In addition, the GzmA inhibitor sepinb6b that completely blocked mouse GzmA activity (Figure 7B), abrogated the expression of IL6 in macrophages incubated with GzmA (Figure 7A), confirming that potential contaminants in GzmA preparations like cathepsin C or traces of LPS were not responsible of the inflammatory effect of extracellular GzmA.

Next we analysed the potential involvement of TLR4 in this process. TLR4 is a key receptor activated by microbial PAMPs like LPS and different endogenous ligands or DAMPs 27 and it has been previously found to be involved in abdominal sepsis 28.

Thus, the effect of extracellular GzmA on the generation of IL-6 and TNFα by M1 bone marrow-derived macrophages from WT and TLR4 deficient mice was analysed. We found that recombinant mGzmA produced in *E. coli* or in *P. pastoris* induced the expression of IL-6 and TNFα on M1 WT macrophages which was reduced in absence of TLR4 (Figure 7C). This result indicates that GzmA induces the expression of IL-6 by a mechanism dependent on TLR4.

It has been reported that inactive human GzmA potentiates the proinflammatory effects of LPS in monocytes by a TLR4-dependent mechanism 26. However, our results employing the GzmA inhibitor serpinb6b suggest that in our system the generation of proinflammatory cytokines is dependent on the enzymatic activity of mouse GzmA. Thus, we decided to analyse if mouse active GzmA potentiated the effect of LPS on mouse macrophages and the requirement of enzymatic activity for this potential effect. We employed a non-saturating low concentration of LPS (1 ng/mL) and three different concentrations of mGzmA. As shown in figure 7D GzmA inactivated with serpinb6b or inactive proGzmA did not induce IL-6 in macrophages. In addition, the combination of active GzmA, inactive proGzmA or GzmA inactivated with serpinb6b with LPS did not increase the level of IL-6 induced by LPS.

### Extracellular GzmA-mediated IL-6 induction in human monocytes is inhibited by antithrombin III

Finally, we analysed if the results found *in vitro* in the mouse model were reproduced in human cells. As shown in Figure 7E, extracellular human GzmA induced IL-6 production in human monocytes, which was completely inhibited by the GzmA inhibitor antithrombin III (ATIII; serpinC1) in the presence of Heparin. Heparin or ATIII alone were not able to inhibit GzmA-mediated IL-6 production. As shown in Figure F we further confirmed that the presence of Heparin was required for ATIII-mediated inhibition of GzmA enzyme activity 29-31.

## Discussion

In this study we have analysed the contribution of GzmA to abdominal sepsis by investigating the presence of extracellular GzmA in serum from patients with peritoneal sepsis as well as the role of GzmA in the mouse model of CLP. GzmA was found to be elevated in human serum from septic patients. The relevance of these findings was supported by the observation that GzmA deficient mice were resistant to CLP as well as to *E. coli*-induced sepsis. GzmA deficiency reduced inflammation without altering bacterial dissemination in the CLP model. A GzmA inhibitor enhanced survival and reduced inflammation in septic WT mice to a similar degree as observed with genetic deficiency, suggesting therapeutic potential in targeting GzmA in peritoneal sepsis.

One of the advantages of CLP model is that the pathogens are endogenous, mimicking the traumatic injury that leads to peritonitis in humans. In addition, CLP sepsis shows a high degree of similarity with the progression of human sepsis, displaying both the hyper- and hypo-inflammatory responses characteristic of human sepsis 32, 33. It has been shown previously that GzmA might contribute to septic shock induced by LPS 16, 17 or to sepsis induced by single bacterial agents like the mouse pathogen *B. microti 18*, *S. pneumoniae* 19 or *E. coli* 34. However, the role of GzmA in abdominal polymicrobial sepsis had so far not been explored. Most importantly, the effect of GzmA inhibition in septic mice had not been analysed in any of these models. Our study thus adds to previous literature and confirms that GzmA is a key mediator of sepsis associated with different bacterial pathogens.

Our data also suggest that inflammation induced by GzmA plays a critical role in the development of sepsis during peritonitis. We observed lower production of IL-1α, IL-1β, IL-6 and TNFα in serum and in peritoneal fluids in GzmA deficient mice. All these cytokines play an important role in sepsis 35-38. In addition, our results employing an inhibitor of extracellular GzmA, serpinb6b, *in vitro* and *in vivo*, strongly suggest that GzmA enhances the inflammatory pathological response in sepsis in the extracellular space. Thus, since serpinb6b should not affect intracellularly perforin-delivered GzmA, we suggest that GzmA does not contribute to sepsis by promoting pyroptosis 39 or other types of cell death 40. This suggestion is further supported by our results showing increased levels of extracellular GzmA in serum from patients with abdominal sepsis. Similar findings have been reported in other septic patients with human bacteraemia, tuberculosis and typhoid fever where high serum levels of GzmA have been detected 41-43 suggesting that this protease may have extracellular effects during other infectious diseases. But how does this protease regulate inflammation from the extracellular milieu?

An important aspect to answer this question is the cell source of GzmA during sepsis. Here we have found that GzmA expression is increased in NK cells during CLP, supporting previous results in other models of sepsis 18, 34. However, this finding does not exclude that GzmA expressed by other cell sources like platelets 23 or other cells 5 could contribute to polymicrobial abdominal sepsis.

We describe here that active GzmA induces the expression of TNFα and IL-6 by a mechanism dependent of both its catalytic activity and TLR4. Supporting our findings in mouse macrophages, a recent study has found that human platelets acquire GzmA expression during aging and induce inflammation in human monocytes, a process inhibited by a TLR4 antagonist 23. Confirming this finding, we have also shown that GzmA induces IL-6 generation in human monocytes, which, similarly to mouse GzmA, was inhibited by SerpinC1/ATIII. Thus, it seems that both mouse and human extracellular GzmA regulates inflammation in monocytes and macrophages by a TLR4 dependent mechanism. However, it is not known yet if GzmA directly activates this receptor or could act on some other substrates that then act as ligands for TLR4. For example, GzmA can cleave fibronectin 44, and fibronectin fragments can activate TLR4 and induce inflammation 45. Since extracellular GzmA induces the generation of IL-6 and TNFα in macrophages in the absence of cell death 46, it is unlikely that TLR4 is activated by the release of inflammatory mediators as a consequence of GzmA-mediated cell death. In addition, several lines of experimental evidence indicate that TLR4 is not activated by contaminants present in the protease preparations. Inactivated GzmA was not able to induce inflammatory cytokines in mouse macrophages or human monocytes, and inflammation was similarly triggered by GzmA generated in *E. coli* or in *P. pastoris* both of which were not contaminated with LPS. In addition, it has also been shown by others that GzmA generated in human cells induces inflammation via a TLR4-dependent mechanism 23*.*

Under normal conditions protease activity in blood is tightly regulated by extracellular inhibitors. Two circulating extracellular inhibitors are known for GzmA, antithrombin III (serpinC1) and α2-macroglobulin 47, suggesting that the protease activity can be regulated at the extracellular level. Interestingly, antithrombin III levels are markedly reduced in sepsis due to the reduction in liver synthesis, the consumption of this protein by the formation of thrombin-antithrombin complexes and by its degradation mediated by neutrophil-released elastase 48. In addition, it has also been observed that in patients with sepsis the levels of α2-macroglobulin are decreased 49. Therefore, supporting our findings it is possible that in sepsis, as natural GzmA inhibitors decrease, the active fractions of this protease in the bloodstream increase, explaining why extracellular GzmA remains active during sepsis. It has been reported that human GzmA potentiates LPS induced cytokine responses in human monocytes, an effect independent of the catalytic activity of GzmA 26. In our study employing mouse GzmA in mouse macrophages and human GzmA in human monocytes, we have found that enzyme activity is required for the induction of IL6 and TNFα. In addition, in our system, active or inactive GzmA did not potentiate the effect of LPS. Other studies employing inhibitors or enzymatically inactive mutants have also found that enzyme activity is required for the inflammatory action of mouse and human GzmA 16, 50. While the reasons for these apparently contradictory results are not known, it cannot be ruled out that both active and inactive forms of GzmA act as proinflammatory mediators in the plasma of septic patients.

In conclusion, using GzmA deficient mice and direct inactivation of GzmA, we have demonstrated that extracellular GzmA plays an important role in sepsis pathogenesis. Other anti-inflammatory treatments including cytokine or TLR4 inhibitors have failed to improve sepsis survival in clinical trials 51, 52. However, a common limitation of those treatments is their potential impact on microbial control and, in addition, they could enhance the hypoinflammatory immunosuppressive stage. Thus, the beneficial effect of reducing inflammation might be counteracted by increasing the persistence of the pathogen(s) responsible for sepsis and/or by enhancing the susceptibility to second infections. Taking into account that GzmA deficiency does not affect pathogen control (our own results and 5), our combined results in human and mouse models suggest that extracellular GzmA is a promising target to treat peritoneal sepsis.

## Materials and Methods

### Mouse Strains

Inbred C57BL/6 (WT) and granzyme A deficient mouse strains (*GZMA*^-/-^) were maintained at the Biomedical Research Centre of Aragon (CIBA). GZMA^-/-^ mice are bred in our animal house employing mice originally provided by Markus Simon´s laboratory. Their genotypes were periodically analysed as described 53. Mice of 8-12 weeks of age were used in all the experiments. Animal experimentation was approved by the Animal Experimentation Ethics Committee of the University of Zaragoza (number: PI43/13).

### Patients and study design

A total of 10 patients with a diagnose of peritonitis and with a SOFA ((Sepsis related Organ Failure Assessment) score ≥2 were prospectively recruited over a 3-month period in 2019 after admission to Lozano Blesa Hospital in Zaragoza, Spain. Blood samples were collected at diagnosis (time 0) and during the first 72 hours. Blood samples were spun down at 2000 xg for 10 min and serum was collected and stored at -80 °C. GzmA concentration in serum was determined by ELISA.

### Cecal Ligation and Puncture

Polymicrobial sepsis (PMS) was induced by CLP performed according to general guidelines 54. WT and *GZMA*^-/-^ mice were anesthetized using 2% isoflurane in oxygen. After disinfecting the abdomen, a 1 cm midline incision was performed to expose the cecum. From the tip, 1 cm of the cecum was ligated using a non-absorbable suture 3-0 (Silkam black 3/0 HS26, Braun) and subsequently perforated twice by a through-and-through puncture with a 20G needle. After gently squeezing the cecum, to extrude sufficient amount of faeces from the perforation, the cecum was returned to the peritoneal cavity and the abdominal musculature was sutured with absorbable suture 3-0 (Novosyn violet 3/0 HR26, Braun). The skin was then sutured with non-absorbable suture 3.0 and a subcutaneous dose of 0.05 mg/kg of buprenorphine in 1 mL of normal saline was administered. After 6 h of the intervention a mixture of antibiotics, ceftriaxone (30 mg/kg) + Metronidazole (12.5 mg/kg), was administered i.p. (intraperitoneal) once a day for 5 days. WT and *GZMA*^-/-^ sham operated mice underwent the same procedure but without CLP.

Mouse survival was monitored for 14 days. Mice were observed three times a day and if there were signs of respiratory distress, pain when touching, vocalisations, hunched posture or inability of a supine animal to stand, the human endpoint was applied.

### *E. coli* sepsis induction

The most frequent Gram-negative bacteria, an *E. coli* strain, was isolated from blood of WT mice after 24 h of sepsis induced by CLP. Bacteria was stored at -80 °C in Luria-Bertani medium (LB; sigma) with 10% glycerol. The inoculum for sepsis induction was prepared from 10 μL of *E. coli* cultured in LB medium at 37 °C to exponential growth phase and washed twice with phosphate-buffered saline (PBS). Absorbance at 600 nm was measured in a spectrophotometer to estimate the number of bacteria in the culture. The density of bacteria was adjusted to 1x10^9^ bacteria/mL. Sepsis was induced in WT, GZMA^-/-^, and GZMA (^+/+^, ^-/-^) littermate mice by i.p. of 2x10^8^ bacteria in 200 μL of PBS.

Mouse survival was monitored for 5 days. Mice were observed three times a day and if there were signs of respiratory distress, pain when touching, vocalisations, hunched posture or inability of a supine animal to stand, the human endpoint was applied.

### Collection of blood, peritoneal lavage fluid and organs

After 6 and 24 h of sepsis induction, a group of septic WT and *GZMA*^-/-^ mice as well as sham operated mice were sacrificed and blood samples were obtained aseptically by cardiac puncture. Anticoagulated blood was kept on ice until further processing for bacteriologic analysis. The rest of blood samples were centrifuged at 2000 xg for 15 min, and plasma was recovered and stored at -80 ºC. To collect peritoneal lavage fluid, 5 mL of sterile PBS was slowly injected into the peritoneal cavity with a 18G needle. The abdomen was gently massaged and then the peritoneal fluid was recovered. Part of it was used for bacteriological analysis while the rest was centrifuged at 2000 xg for 10 min and the supernatant was stored at -80 ºC. Finally, spleen, liver and lungs were collected aseptically, homogenized in 1 mL of PBS and used for bacteriological analysis.

### Aerobic bacterial count and characterization by MALDI-TOF mass spectrometry

Peritoneal lavage fluid, blood and homogenized organs were serially diluted in PBS and plated by duplicate onto MacConkey and Columbia CNA agar (Biomériux), which were incubated aerobically for 48 h at 37 °C. Plates that contained between 30 and 300 colonies were counted and the number of CFU was determined. Aerobic bacterial isolates were identified by MALDI-TOF mass spectrometry.

### Infection of bone marrow macrophages with *E. coli*

M2 macrophages were differentiated from mouse bone marrow (BM). Cells were aseptically collected form BM and resuspended in RPMI medium supplemented with 10% of FCS serum, 100 U/mL of penicillin/streptomycin, 50 mM of 2-ME, and 10% supernatant of L-929 cell culture as source of M-CSF. Cells were seeded at a density of 1 x 10^6^ cells/mL and allowed to differentiate for 6 days at 37 °C and 5% CO_2_ atmosphere. For *in vitro E. coli* infection experiments, 5 x 10^5^ macrophages were seeded in 96 well plates, incubated with 300 nM of active GzmA and infected with *E. coli* at a MOI of 1:100 for 1 h at 37 °C and 5% CO_2._ Subsequently, medium was removed, cells were washed with PBS and further incubated with complete RPMI medium containing 30 μg/mL of gentamycin for 1 h. Cells were washed and incubated with complete RPMI medium for 4, 24 and 48 h. After incubation periods, the macrophages were lysed with Triton-X 0.1% and CFU number was determined.

### Inflammation induced by GzmA in bone marrow derived macrophages

M1 Macrophages were differentiated from WT and TLR4^-/-^ mouse bone marrow. Cells were aseptically collected from femurs and tibias and 1 x10^6^ cells were cultured on 100 mm petri dishes with 10 mL of RPMI 1640 medium containing 10% of FCS serum, 100 U/mL of penicillin/streptomycin, 50 mM of 2-ME, and 10% of supernatant of X63Ag8653 cell cultures as source of GM-CSF 55 (complete medium). On days 3 and 5, the supernatant was removed and 10 mL of fresh complete medium was added. On day 7, macrophages showed a complete differentiated phenotype expressing CD11b and F4/80. WT and TLR4^-/-^ macrophages were stimulated with active GzmA (300 nM), GzmA inactivated with the inhibitor serpinb6b (2,4 µM), *E. coli*-derived LPS (100 ng/mL) or heat killed *S. aureus* (*S. aureus* HK,1 x10^8^ CFU/mL). In some experiments, LPS, GzmA, serpinb6b and cathepsin C (Sigma) were added at different concentrations. After 24 h of incubation, the supernatant was collected to determine the cytokine levels by ELISA.

### Inflammation induced by human GzmA in monocytes

Monocytes were isolated from peripheral blood of healthy donors by a ficoll gradient centrifugation. Diluted blood was carefully layered on top of 15 mL of Histopaque ®-1077 (1.077 g/mL) (Sigma) solution. Centrifugation was performed for 10 min at 1455 xg without brake. After centrifugation, the white ring of peripheral blood monuclear cells (PBMCs) were collected, resuspended in 15 mL of complete DMEM medium (10% of FCS serum, 100 U/mL of penicillin/streptomycin, 50 mM of 2-ME), placed on a 75 cm^2^ flask and incubated for 2 h at 37 °C and 5% CO_2_ to allow monocytes to adhere at the flask bottom. After 2 h of incubation, cells were washed with PBS to remove cells in suspension. Adherent cells were trypsinized and 5 x 10^5^ monocytes were seeded in 96 well plates and incubated for 2 h to allow them to adhere. Monocytes were stimulated with LPS (100 ng/mL), human GzmA (300 nM), antithrombin III (8.6 μM), and heparin (430 μM). After 24 h of incubation, supernatant was collected to determine IL-6 by ELISA.

### Recombinant GzmA expression from *Pichia pastoris*

*P. pastoris* expressing mouse proGzmA was grown at 30 °C for 36 h, and then allowed to settle for 12 h at room temperature; growth medium was replaced with induction medium containing 3% methanol and 0.5 M arginine. Induction was performed at 23 °C for 60 h. Culture was centrifugated, the supernatant was collected and then filtered. Recombinant proGzmA was purified by cation exchange chromatography. Active GzmA was obtained using cathepsin C which hydrolyse the N-terminal dipeptide present in proGzmA. After activation, cathepsin C was not removed and active GzmA was stored at -80 °C until further use.

### Recombinant GzmA expression from *Escherichia coli*

Mouse proGzmA was cloned into pET21a and transformed into *E. coli* BL21. For protein production, *E. coli* expressing proGzmA were grown at 37 °C until the culture reached an optical density at 600 nm between 0.6 and 0.8. The protein expression was induced adding IPTG 1 mM and incubating for 3 hours at 37 °C. The culture was centrifugated at 8300 xg for 15 min. The supernatant was discarded and the pellet was resuspended in lysis buffer (Tris-HCl 20 mM, NaCl 100 mM, DTT 2 mM, lysozyme 2 mg/mL, DNAse 1 mg/mL and protein inhibitor). Bacteria was lysed by sonication, centrifugated at 48400 xg for 15 min at 4 °C and the supernatant was discarded. Previously obtained inclusion bodies were resuspended in buffer denaturation (100 mM Tris-HCl, 6 M GuCl_2_, 20 mM EDTA and 10 mM oxidized DTT) and then centrifugated at 48400 xg for 15 min at 4 °C. Proteins were refolded in 100 volumes of refolding buffer (100 mM Tris-HCl, 500 mM arginine, 10% glycerol and 10 mM oxidized DTT) for 3 days at 4 °C and then dialyzed five times in MT-PBS. Recombinant proGzmA was purified by cation exchange chromatography. Active GzmA was obtained using cathepsin C which hydrolyse the N-terminal dipeptide present in proGzmA. After activation, active GzmA was stored at -80 °C until further use. Cathepsin C was inactivated by increasing pH to 7.4 and it was confirmed that it did not induce the generation of inflammatory cytokines in macrophages.

### Recombinant mouse serpinb6b expression from *Pichia pastoris*

*Pichia pastoris* expressing serpinb6b was kindly provided by Phil Bird from Monash University, Australia. *P. pastoris* expressing serpinb6b was grown at 30 °C for 36 h and then allowed to settle for 12 h at room temperature; growth medium was replaced with induction medium containing 3% methanol and 0.5 M Arginine. Induction was performed at 23 °C for 60 h. Culture was centrifuged and a chemical and physical lysis was performed. After supernatant clarification, recombinant serpinb6b was purified by immobilized metal (Nickel) affinity chromatography.

### LPS quantification in mouse and human GzmA preparations

Mouse and human proGzmA LPS quantification was carried out using the Toxin Sensor™ Endotoxin Detection System (GenScript). Samples were treated and analyzed following the manufacturer instructions. The amount of LPS in all protein preparations was always lower than 0.5 EU/μg protein.

### Determination of GzmA and cytokine levels

Levels of mouse IL-1α, IL-1β, TNFα and IL-6 were measured in peritoneal fluid and plasma, 6 and 24 h after CLP induction using commercial ELISA kits (Invitrogen). Human serum GzmA levels from healthy donors and patients with confirmed abdominal sepsis at diagnosis and during the first 72 h was monitored by a commercial ELISA kit (Human Granzyme A DuoSet ELISA, R&D Systems) following the manufacturer recommendations.

### Isolation of Mouse Peripheral Blood Lymphocytes (PBLs)

After 24 h of sepsis induction, WT and *GZMA*^-/-^ mice were sacrificed. Total blood was collected aseptically by cardiac puncture in presence of anticoagulants Sodium Citrate 3.8%. PBLs were isolated by gradient centrifugation. 1 mL of total blood was mixed with 1 mL PBS. To a conical 15 mL tube, 2 mL of Histopaque-1077® was added and then blood was carefully layered on top. Next, the sample was centrifuged at 400 xg for 30 min at room temperature. Mononuclear cells were collected from the opaque interface.

### Intracellular expression of GzmA

After 24 h of sepsis induction, WT and *GZMA*^-/-^ mice were sacrificed. Blood and spleen were collected aseptically. PBLs were isolated from blood as described above and spleen was homogenized in 5 mL of RPMI medium. 2x10^5^ PBLs or splenocytes were stained with anti CD3-FITC, anti CD8-APC, anti NK1.1-APC-Vio770 and anti CD4-VioBlue from Miltenyi Biotec. Subsequently, cells were fixed with paraformaldehyde (PFA) 1%, permeabilised with saponin 1% in PBS and incubated with anti gzmA-PE (eBioscience) or with the corresponding isotype control (IgG2b kappa isotype control PE, eBiosciencie). Finally, intracellular expression of GZMA was analysed by FACS.

### Therapeutic inhibition of extracellular GzmA with Serpinb6b

After sepsis induction a group of WT and *GZMA*^-/-^ mice were treated with 40 µg of the GzmA inhibitor serpinb6b i.p. The inhibitor was administrated after 6 h and once a day for 5 days combined with ceftriaxone (30 mg/kg) + metronidazole (12.5 mg/kg). Survival was monitored for 14 days.

## Figures and Tables

**Table 1:** Clinical data of abdominal sepsis patients.

Patient	SOFA score	Gender	Age	Status at day 30	Co-morbidities
1	2	Male	62	Alive	Gastric tumor + chemotherapy
2	2	Male	68	Alive	Coronary disease, peripheral vascular disease, peptic ulcer
3	5	Female	75	Dead	Dementia, cerebrovascular disease, renal disease, diabetes with damage to target organs
4	7	Female	72	Alive	Congestive heart failure, connective tissue disease (immunosuppression), renal disease
5	7	Male	75	Alive	Diabetes, bile-duct tumor
6	8	Male	67	Alive	Coronary disease, congestive heart failure, diabetes with damage to target organs
7	6	Female	78	Dead	None
8	6	Male	90	Alive	None
9	7	Female	82	Dead	Cerebrovascular disease, peptic ulcer, renal disease, renal tumor
10	9	Male	94	Alive	Chronic pulmonary disease
